# Successful Preoperative Radiotherapy for Neglected Shoulder Liposarcoma: A Retrospective Case Report

**DOI:** 10.1155/2024/5782352

**Published:** 2024-08-27

**Authors:** Camilla Linhart, Craig MacLeod

**Affiliations:** ^1^ Doctor of Medicine (MD) University of Notre Dame Australia (Sydney Campus), Darlinghurst, New South Wales 2010, Australia; ^2^ Border Medical Oncology GenesisCare Albury Wodonga Regional Cancer Centre, Albury, New South Wales 2640, Australia

**Keywords:** liposarcoma, radiotherapy, shoulder

## Abstract

This study examines a unique case of a 61-year-old male with a 5-year history of a progressively growing mass above his right shoulder, diagnosed as a dedifferentiated pleomorphic liposarcoma. Using computerized tomography-guided core needle biopsy, the tumour was identified as intermediate to high grade. Surgical removal required preoperative radiotherapy to reduce the size of the tumour. Several unique characteristics set apart this particular case of liposarcoma: its substantial size, its unpredictable growth pattern, its absence of metastasis, and notably, its prolonged period of being untreated. This case report outlines the clinical background, diagnostic procedures, and treatment modalities employed in managing this condition, emphasizing a localized dual therapy approach combining radiotherapy and surgery. Emphasis is placed on distinguishing liposarcoma from lipoblastoma, a benign adipocyte tumour, to facilitate accurate diagnosis and appropriate treatment selection. The positive result achieved in this case could provide valuable insights for the future treatment and management of similarly sized aggressive tumours.

## 1. Introduction

Liposarcoma, a rare malignant tumour arising from adipose tissue in the body's soft tissues, is the most prevalent adult soft tissue sarcoma, constituting less than 17% of all cases [1]. Typically presenting around the age of 60 with an equal incidence between genders, liposarcoma manifests predominantly in the limbs, especially the thigh, inguinal area, and retroperitoneal regions like the abdomen [2]. Disease severity varies based on subtype, presentation stage, and primary tumour location [1]. The exact cause remains elusive, with studies suggesting genetic mutations within adipocytes' DNA leading to unregulated growth. Potential risk factors include prior radiation, familial cancer syndromes, lymphatic system damage, and prolonged exposure to specific chemicals such as vinyl chloride [1].

Patients with liposarcoma are often asymptomatic until the tumour becomes sizable, causing tenderness, pain, or functional disturbances; abdominal enlargement may occur in abdominal cases [1, 2]. Skin liposarcomas exhibit an exophytic growth pattern, presenting as dome-shaped or polypoid lesions primarily centered in the dermis [1].

The World Health Organization classifies liposarcomas into five categories: well-differentiated, undifferentiated, pleomorphic, dedifferentiated, myxoid, and round cell [2, 3]. The prognosis varies significantly, with well-differentiated liposarcomas having a 99% 5-year survival rate, while undifferentiated and dedifferentiated pleomorphic liposarcomas have a high recurrence rate and poor overall prognosis [1, 2, 4, 5].

The magnetic resonance imaging (MRI) and CT with whole-body fludeoxyglucose (FDG) positron emission tomography (PET) scan are instrumental in locating and staging liposarcoma; additionally, CT-guided core needle biopsy and fluorescence in situ hybridization (FISH) diagnose the sarcoma pathologically and genetically, respectively, further facilitating treatment planning. Surgical intervention, tailored to tumour size and location, is augmented by local radiation and/or chemotherapy for high-grade lesions to mitigate recurrence. Given liposarcoma's propensity for recurrence over months to years, diligent posttreatment monitoring is imperative [4].

We present a new case of primary shoulder liposarcoma and provide a review of the relevant literature. This case report complies with NHMRC guidelines, having received ethics approval from both St Vincent's Sydney HREC and the UNDA Human Research Ethics Committee (reference 2023-087). Patient data was collected by Dr. Craig MacLeod, and student doctor Camilla Linhart accessed and analysed the data from GenesisCare Mosaiq at the Albury Cancer Centre, Australia. We extend our sincere thanks to radiation therapist Emily Evans from Albury Wodonga GenesisCare for acquiring the data, as well as to the Victorian Cancer Cytogenetics Department and the Histopathology Department at St Vincent's Hospital Melbourne for their valuable contributions.

## 2. Case Report

A 61-year-old previously healthy male, weighing 100 kg, presented with a 5-year history of a progressively enlarging mass superior to his right shoulder, displaying a notable acceleration of growth in the last 2 years. The patient reported no associated pain and maintained the function of his right shoulder and arm. Noteworthy is the patient's familial predisposition to cancer, with his mother succumbing to metastatic melanoma at 74 and his father to lung cancer at 64. After being referred to the Albury-Wodonga Cancer Centre, GenesisCare, the patient underwent a clinical review in May 2022.

During the physical examination, a pedunculated mass was identified at the apex of the patient's right shoulder, positioned over the acromioclavicular and glenohumeral joint spaces. This superficially located mass was characterized by a distinct “light bulb” morphology with a larger head and a smaller pedicle base, exhibited no breach of the skin, was nontender, and lacked signs of warmth or erythema. Despite the mass, the patient maintained good overall health, remained afebrile, and exhibited stable vital signs. Laboratory investigations and inflammatory markers yielded results within the normal range.

A CT-guided core needle biopsy conducted in June 2022 confirmed the presence of an intermediate to high-grade liposarcoma. Subsequent MRI examination delineated a large, well-circumscribed, encapsulated mass measuring approximately 147 × 192 × 135 mm, with a volume of 2944 mL superior to the right shoulder girdle. The heterogenous mass exhibited T1 hyperintense central regions and internal septation and showed no invasion into underlying muscle or bone. Staging procedures, including a PET scan of the right shoulder, revealed avidity in the primary tumour with no evidence of metastatic disease as lymph nodes and organs remained clear.

To facilitate surgical excision of the mass, the patient underwent preoperative radiotherapy from July to August 2022, receiving a total dose of 50.4 Gy in 28 fractions. The objective of this intervention was to significantly mitigate the risk of locoregional recurrence and to diminish the size of the mass. Following 28 consecutive days of radiation treatment, the liposarcoma demonstrated a reduction in dimensions to 120 × 100 mm and to a volume of 2064 mL. Thus, a 30% (879 mL) reduction in tumour volume makes it more amenable to surgical excision.

In October 2022, surgeons at St. Vincent's Hospital in Melbourne effectively excised the liposarcoma. The medical team observed a favourable reaction to preoperative radiotherapy and confirmed the surgery's success, achieving clear margins around the tumour. Wide surgical margins were established to deter recurrence and cellular dedifferentiation [2].

## 3. Diagnostic Tests

**Figure 1:**
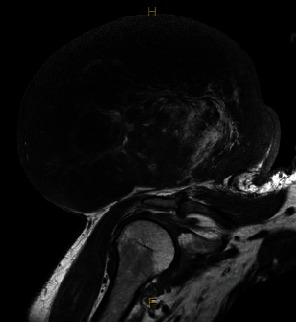
MRI of right shoulder: preradiotherapy treatment in the frontal plane.

**Figure 2:**
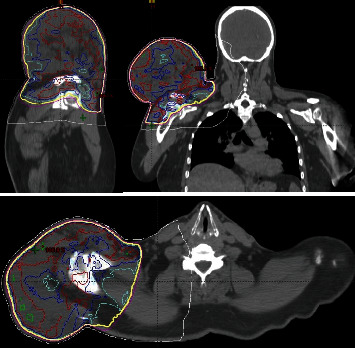
CT planning of tumour in sagittal, frontal, and transverse planes.

**Figure 3:**
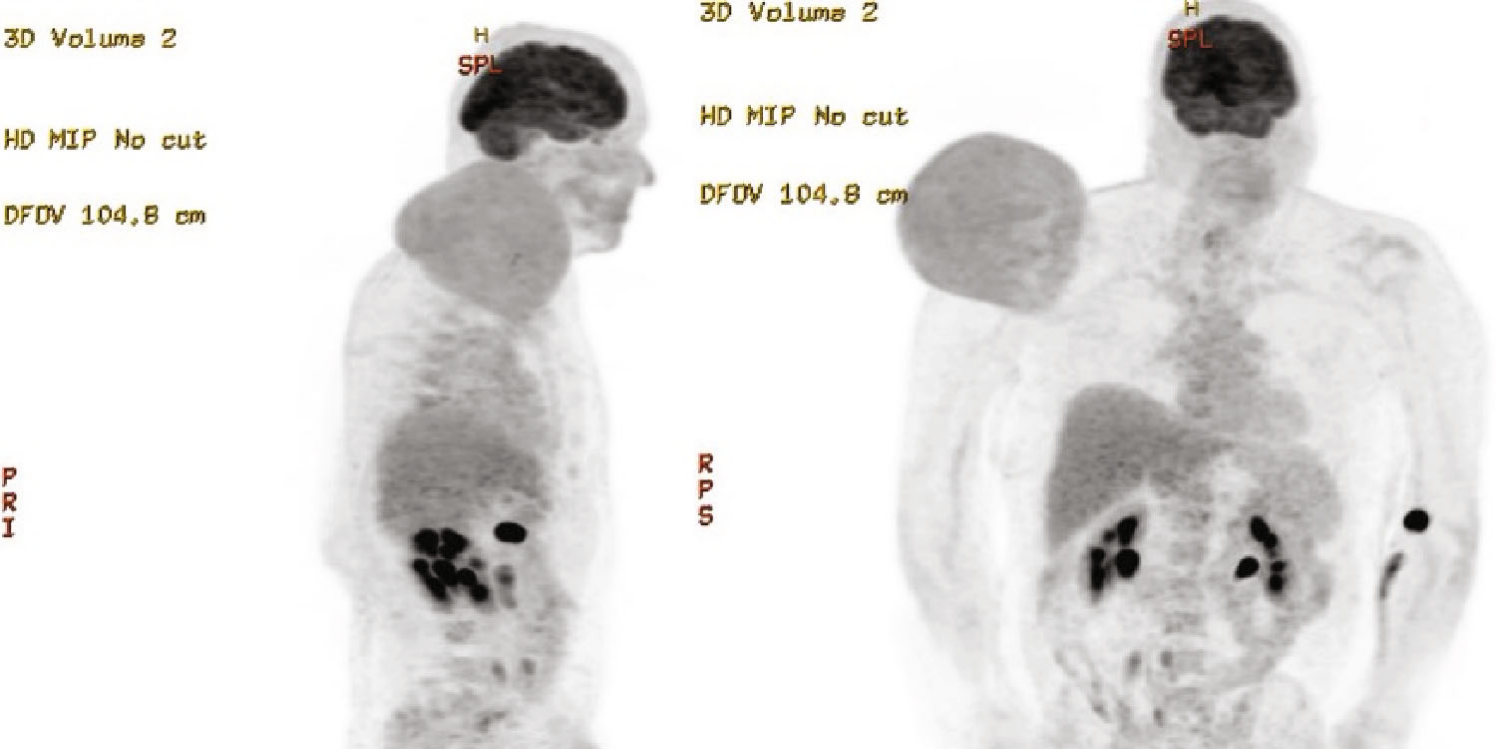
Tumour staging via FDG-PET in sagittal and frontal planes.

The contrast-enhanced MRI (Figure 1) revealed a sizable, well-defined, encapsulated mass superior to the right shoulder girdle, measuring 147 × 192 × 135 mm (AP x ML x SI). The mass exhibited a predominantly heterogenous T1 hypointense signal, featuring central regions with a T1 hyperintense, fat-intensity signal. Internal septations and nodular margins were evident throughout the lesion, with no infiltration observed in adjacent muscular or osseous structures. Despite the relatively limited adipose tissue volume, the mass raised concerns for an intermediate to high-grade liposarcoma. Staging using CT (Figure 2) with whole-body FDG PET (Figure 3) indicated a mildly metabolically active liposarcoma, with no evidence of metabolically active metastatic disease.

## 4. Pathology

**Figure 4:**
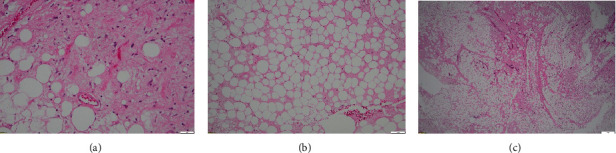
Haematoxylin-eosin stain. (a) ×400 magnification. Viable tumour composed of variably sized adipocytes and scattered pleomorphic spindle cells in fibromyxoid stroma. (b) ×100 magnification. Tumour includes necrotic area with reduced cellularity. (c) ×40 magnification. Dedifferentiated liposarcoma showing discrete areas of nonadipocytic sarcomatous components (stained pink).

The histopathology characterized the mass as a well-circumscribed fleshy tumour with central necrosis and abscess. The peripheral aspect exhibited a myxoid glistening appearance, while the central region displayed yellow necrosis with a 100 mm diameter cystic cavity containing yellow exudate, constituting approximately 60% of the tumour. The pathological diagnostic criteria for dedifferentiated liposarcoma include identifying a transition from an atypical lipomatous tumour/well-differentiated liposarcoma to a nonlipogenic sarcoma with variable histological grades [6]. Microscopic analysis revealed areas of central necrosis surrounded by viable tumour tissue. The surrounding regions contained fat tissue fragments with adipocytes of varying sizes, scattered spindle cells within a fibromyxoid stroma, and branching thin-walled blood vessels. Although occasional mitotic figures and scattered mononuclear inflammatory cells were observed, there was an overall reduction in cellularity by%–50-60% (Figure 4). Clear surgical margins were noted, with the deep margin measuring 5 mm and consisting of deep fascia and a fibrous capsule.

T cell, B cell, and NK cell lymphoid markers were tested, but insufficient lymphocytes were available for flow cytometric analysis. Cytogenetic analysis may be of value when diagnosing lipomatous tumours because different tumour subtypes have different chromosomal abnormalities [2]. FISH was conducted to delineate myxoid molecular cytogenetic markers, including the *DDIT3* gene (formerly known as *CHOP*) rearrangements on chromosome 12q13.3 and the *FUS* gene on 16p11.2. These markers are indicative of the t(12; 16) (q13.3; p11.12) translocation, a hallmark observed in approximately 90% of myxoid liposarcoma cases.

**Figure 5:**
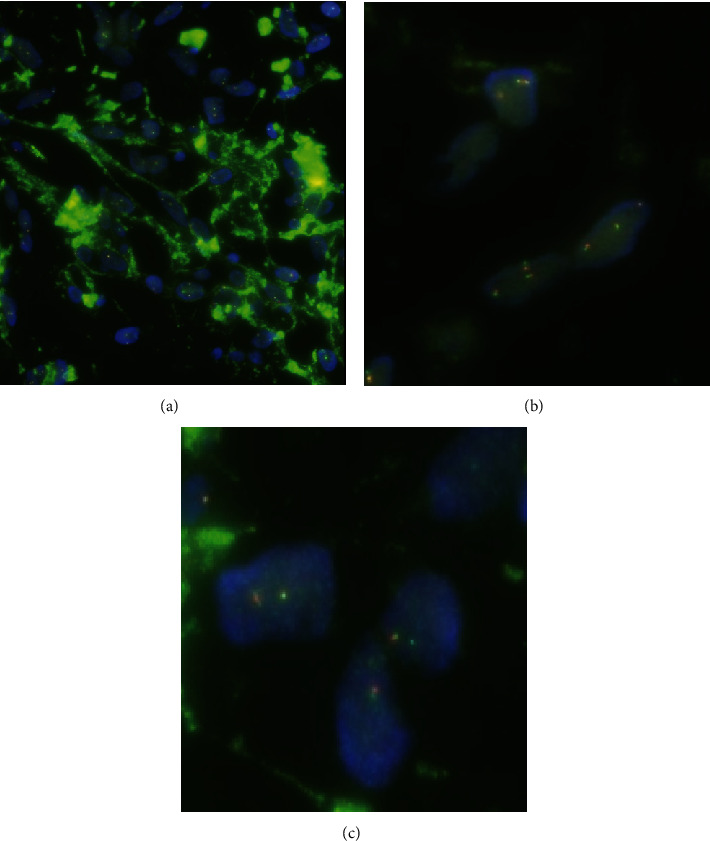
Fluorescence in situ hybridization. The CytoOrange fluorochrome signals are the 3′ end of DDIT3 and the CytoGreen signals are the 5′ end of DDIT3 gene. (a) Demonstrates duplication of DDIT3 gene in low level. (b) DDIT3 break-apart probe (MetaSystems) showing evidence of gain of DDIT3 but no evidence of gene rearrangement. (c) FUS break-apart probe (MetaSystems) showing no evidence of FUS gene rearrangement.

In this patient's case, FISH analysis revealed a gain of the *DDIT3* gene, resulting in three copies within the 12q13.3 region (Figure 5). This observation is specific to the segment of the long arm (*q*) of chromosome 12, leading to a trisomy rather than the typical disomy. Notably, no *DDIT3* or *FUS* gene rearrangements were detected, prompting the classification of the sarcoma as a dedifferentiated liposarcoma. The implications of such a genetic duplication hinge upon the genes residing within the duplicated region. Duplication may culminate in the heightened expression of these genes, potentially instigating developmental anomalies, growth aberrations, or facilitating the pathogenesis of certain malignancies. Indeed, in this instance, the occurrence of liposarcoma underscores the contributory role of gene duplication to oncogenesis.

## 5. Treatment

**Figure 6:**
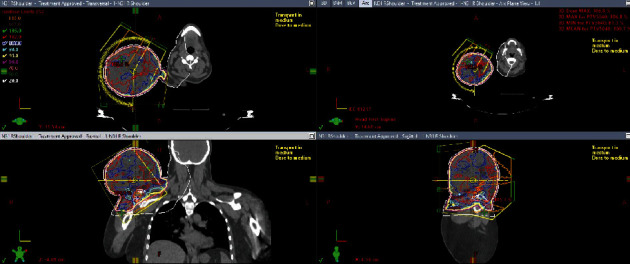
Radiotherapy treatment plan using VMAT.

**Figure 7:**
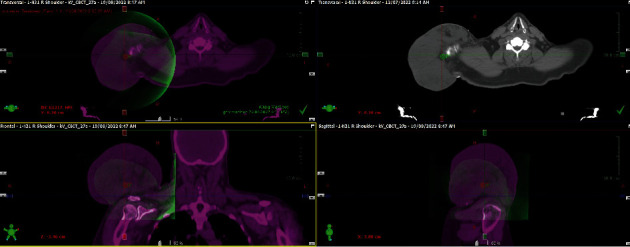
Postradiotherapy treatment field (Inner green area indicates tumour shrinkage after 28 fractions of RT).

The patient underwent daily preoperative radiotherapy from July 14 to August 22, 2022, using volumetric arc therapy (VMAT) on a linear accelerator (LINAC) (Figure 6). The prescribed treatment consisted of 50.4 Gy in 28 fractions (1.8 Gy/fraction) with 6 MV photons, complemented by daily cone beam CT for precise tumour position correction (Figure 7). Common inflammatory side effects, including erythema, swelling, and localized pain, were observed, with the patient tolerating the treatment well. Within the first 3 weeks, the patient reported bleeding, severe fatigue, a fever, and a 5-kg weight loss. Blood results indicated leucocytosis, neutrophilia, an elevated CRP of 161 mg/L, and hyperglycaemia, suggestive of a localized sarcoma site infection and diabetes mellitus type 2. Treatment included IV cephazolin, paracetamol, oxycodone, metformin, and insulin. Pathology of the wound swab revealed moderate growth of beta-haemolytic streptococcus pneumoniae and light growth of staphylococcus aureus. Xerosis and mild oedema were also noted during treatment.

**Figure 8:**
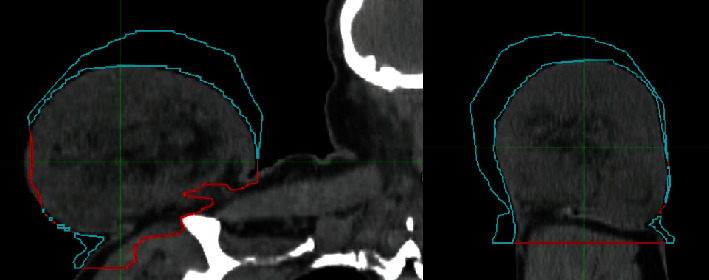
CT of right shoulder postradiotherapy treatment (blue lines indicate tumour volume reduction) in frontal and sagittal planes.

**Figure 9:**
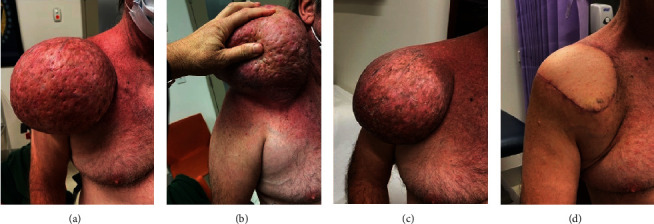
Tumour size before and during (a–c) RT and (d) shoulder postsurgery.

Seven weeks postradiotherapy, skin inflammation subsided, and the mass measured 120 × 100 mm, a 30% reduction in tumour volume (Figure 8). Tumour excision and plastic surgery were performed in October 2022 at St Vincent's Hospital in Melbourne, with the patient being a candidate for an ipsilateral latissimus dorsi flap. The surgery proceeded without complications (Figure 9).

## 6. Discussion

The rarity of primary liposarcomas of the shoulder is evident, with only 11 reported cases documented since 1994, as indicated in Table 1. A literature review conducted on June 15, 2023, revealed a mean age of 76 years for 10 of these cases. In this context the occurrence in a 2-year-old patient initially diagnosed with a right shoulder liposarcoma was unusual. It was later identified as a lipoblastoma, a common paediatric soft tissue neoplasm [15].

Liposarcomas and lipoblastomas are differentiated diagnostically through advanced molecular and imaging techniques. These sophisticated methods, which reveal chromosomal rearrangements and distinctive signal intensity patterns, encompass advanced cytogenetic FISH analysis and MRI, respectively [15].

Chromosomal abnormalities leading to fusion proteins play a crucial role in mesenchymal cancer development [2]. In lipoblastomas, FISH identifies chromosomal rearrangements in the 8q11-13 region, specifically targeting the *PLAG1* gene on Chromosome 8 [16]. In contrast, liposarcomas exhibit FISH-identified chromosomal translocations involving Chromosomes 12 and 16, t(12:16). This targets the *DD1T3* gene at 12q13.31 and the *FUS* gene at 16p11.2. Notably, the *FUS::DDIT3* fusion gene, a common chromosomal translocation in liposarcoma, encodes a transcription factor essential for adipocyte differentiation [2, 14]. MRI distinguishes between lipoblastomas and liposarcomas based on the signal intensity of the fat component, mature adipose tissue. Lipoblastomas typically exhibit heterogeneous signals, being hyperintense (bright) and hypointense (dark) on T1-weighted images. In contrast, liposarcomas display a homogenous hypointense signal on T1-weighted images. Both tumour types, however, present hyperintense homogenous signals on T2 weighted images [5, 11].

Histologically similar, lipoblastomas and liposarcomas vary in clinical presentation, behaviour, and potential malignancy [5]. Lipoblastomas, typically benign, present as slow-growing, painless nodules confined to the hypodermis in paediatric patients, while liposarcomas, prevalent in adults aged 60 to 80, manifest as larger, potentially metastatic masses with the potential to spread to the lungs or liver [17]. Noteworthy from Table 1 is that the reported shoulder liposarcomas were nontender enlarging masses, with varying diameters between 2 and 9 cm. Among the cases, well-differentiated liposarcomas, the least aggressive subtype, were predominant, and cytogenetic analysis identified dedifferentiated subtypes associated with metastasis [2]. Despite varying treatment approaches, all tumours were excised, with survival rates ranging from 6 to 18 months, emphasizing the importance of multimodal treatment strategies in locally advanced soft tissue sarcomas [12].

The patient's case of dedifferentiated liposarcoma of the right shoulder is notable for its lack of metastasis despite the tumour's substantial size and progressive growth. Dedifferentiated liposarcomas are generally less responsive to radiotherapy alone, necessitating a multimodal treatment approach that includes surgery, radiotherapy, and sometimes chemotherapy. Radiotherapy is typically employed to shrink tumours preoperatively or to eradicate residual cancer cells postoperatively [18]. Wide surgical resection combined with preoperative radiation is recommended for intermediate and high-grade liposarcomas [5]. Oncologist Dr. Xin-Li Wang, based at the International Medical Center Hospital in China, emphasized that chemotherapy serves as the standard systemic treatment for advanced, unresectable, and/or metastatic cases. However, its therapeutic efficacy is often limited, with varied response rates ranging between 3.9% and 20% according to past studies [15].

Cytogenetic analysis in this patient's case unveiled a notable gain of 12q13.3, featuring increased copy number of the *DD1T3* gene. Such genetic aberrations are often associated with amplification of oncogenes like *MDM2* and *CDK4*, which are crucial in the development and progression of well-differentiated and dedifferentiated liposarcomas. However, to determine such amplifications, FISH analysis must be specifically performed for *MDM2* and *CDK4* genes. This was not conducted in this patient's case. Associated heightened tumour aggressiveness and relatively radio-resistance by these types of liposarcomas pose challenges in treatment planning. This genetic profile underscores the imperative of a comprehensive treatment approach, where the synergistic utilization of radiotherapy and surgery assumes pivotal significance in achieving robust local control, as exemplified by the patient's therapeutic trajectory.

The decision against employing conventional chemotherapy as part of a triple therapy approach reflects a deliberate consideration of treatment priorities, especially given the tumour's inherent resistance to chemotherapy. Furthermore, the potential side effects and toxicity associated with chemotherapy underscore the need for a judicious assessment of risks versus benefits in each clinical scenario. Considering the limited evidence regarding the efficacy of chemotherapy in aggressive sarcoma subtypes, the patient's medical team opted for an aggressive localized dual therapy strategy. This decision reflects a concerted effort to prioritize the patient's overall health and quality of life, acknowledging the nuanced balance between treatment efficacy and tolerability in the management of dedifferentiated liposarcoma.

The treatment approach overall in liposarcoma is multimodal and often tailored to the individual patient based on the specific subtype of liposarcoma, the genetic profile of the tumour, the tumour's size and location, and the overall health of the patient. Currently, targeted therapies against *MDM2* and *CDK4* amplifications are under investigation and hold promise for improving future outcomes. Conversely, myxoid liposarcomas with *DDIT3* translocations (*FUS::DDIT3* fusion) generally respond better to radiotherapy, highlighting the variability in treatment responses based on specific genetic profiles. The role of chemotherapy in liposarcoma remains an area of active research and ongoing clinical trials [19].

This case study exemplifies a tumour that has grown significantly over time due to neglect. Existing literature underscores the multifaceted nature of tumour neglect, encompassing factors related to the patient, practitioner, and society. Patient-related aspects involve asymptomatic presentation, challenges in recognizing subtle cancer symptoms like weight loss and nonhealing lesions, low prioritization of medical appointments for abnormal findings, lack of a primary general practitioner, and patient denial. Practitioner-related factors include nonspecific symptoms and delayed intervention. Furthermore, societal factors, such as inadequate public awareness, collectively contribute to delays in diagnosis and tumour progression [20, 21].

This article presents a thorough examination of the specific findings pertaining to a rare case of dedifferentiated liposarcoma. Known for its notable metastatic potential and resistance to radiotherapy, this subtype of tumour poses considerable clinical challenges. However, in the case under study, the absence of metastasis coupled with the tumour's responsiveness to radiotherapy marks a significant success in the dual therapy treatment approach. By offering a comprehensive analysis of this case, the study serves as a valuable reference for guiding future treatment approaches for aggressive liposarcomas. The meticulous dimensions of the research are adeptly explored, providing a nuanced discussion of its clinical implications. Furthermore, the article conscientiously addresses study limitations, acknowledging the hurdles encountered and identifying avenues for further investigation. In particular, the discussion delves into the potential role of fusion protein genetics in sarcoma treatment and the experimental nature of chemotherapy in liposarcoma management. This insightful exploration not only deepens our understanding of the subject but also underscores the imperative for ongoing research into targeted therapies and multimodal treatment strategies.

## Figures and Tables

**Table 1:** Clinical, histologic characteristics, treatment, and outcome of case reports of shoulder liposarcoma from 1994 to 2022.

**Author**	**Study year**	**Patient age (years)**	**Sex**	**Grade**	**Primary tumour size (cm)**	**Histology**	**Metastasis**	**RT**	**CT**	**Surgery**	**Follow-up**
Dei Tos et al. [7]	1994	54	M	Low	7	WDLS	—	—	—	+	aNED after 12 months
Dei Tos et al. [7]	1994	45	M	—	3.2	WDLS	—	—	—	+	Recurrences excised at 8, 9, 12, 14, and 17 years w/o signs of recurrence 3 years later
Dei Tos et al. [7]	1994	35	M	Low	2	WDLS	—	—	—	+	aNED after 24 months
Dei Tos et al. [7]	1994	82	F	—	5	WDLS	—	—	—	+	Died of unrelated disease at 3 years w/o signs of recurrence
Matsumoto et al. [8]	2000	71	M	Low	—	WDLS	—	—	—	+	aNED after 12 months
McElderry, McKenney, and Stack [9]	2008	68	M	High	4.6	DDLS	Gingival mucosa	+	—	+	aNED after 6 months
Peiper [10]	2008	42	M	High	—	MLS	—	+	+	+	—
Al-Tammar [11]	2021	2	M	—	2.3	MLS	—	—	—	+	aNED after 18 months
Wang, Gong, and Xue [12]	2021	61	M	High	5	DDLS	Abdomen + Lungs	—	+	+	aWD after 8 months
Choi et al. [13]	2022	24	F	High	9	PLS	—	+	—	+	aNED after 6 months
Ghieh. Beaineh, and Ibrahim [14]	2022	52	M	Low	—	WDLS	—	—	—	+	—

Abbreviations: aNED, alive, no evidence of disease; aWD, alive with disease; CT, chemotherapy; DDLS, dedifferentiated liposarcoma; MLS, myxoid liposarcoma; PLS, pleomorphic liposarcoma; RT, radiotherapy; WDLS, well-differentiated liposarcoma.

## Data Availability

Since this is a retrospective case study, data sharing does not apply to this article, as no new datasets were generated or analyzed.
